# Florbetapir PET-assessed demyelination is associated with faster tau accumulation in an APOE ε4-dependent manner

**DOI:** 10.1007/s00259-023-06530-8

**Published:** 2023-12-05

**Authors:** Anna Rubinski, Anna Dewenter, Lukai Zheng, Nicolai Franzmeier, Henry Stephenson, Yuetiva Deming, Marco Duering, Benno Gesierich, Jannis Denecke, An-Vi Pham, Barbara Bendlin, Michael Ewers, for the Alzheimer’s Disease Neuroimaging Initiative

**Affiliations:** 1 Institute for Stroke and Dementia Research, University Hospital, Ludwig-Maximilian-University Munich, Munich, Germany; 2 Department of Medicine, School of Medicine and Public Health, University of Wisconsin- Madison, Madison, WI USA; 3 Medical Image Analysis Center (MIAC) and Department of Biomedical Engineering, University of Basel, Basel, Switzerland; 4 Department of Neuroradiology, School of Medicine, Technical University of Munich, Munich, Germany; 5 German Center for Neurodegenerative Diseases (DZNE), Munich, Germany

**Keywords:** Myelin, Florbetapir-PET, Tau, APOE

## Abstract

**Purpose:**

The main objectives were to test whether (1) a decrease in myelin is associated with enhanced rate of fibrillar tau accumulation and cognitive decline in Alzheimer’s disease, and (2) whether apolipoprotein E (APOE) ε4 genotype is associated with worse myelin decrease and thus tau accumulation.

**Methods:**

To address our objectives, we repurposed florbetapir-PET as a marker of myelin in the white matter (WM) based on previous validation studies showing that beta-amyloid (Aβ) PET tracers bind to WM myelin. We assessed 43 Aβ-biomarker negative (Aβ−) cognitively normal participants and 108 Aβ+ participants within the AD spectrum with florbetapir-PET at baseline and longitudinal flortaucipir-PET as a measure of fibrillar tau (tau-PET) over ~ 2 years. In linear regression analyses, we tested florbetapir-PET in the whole WM and major fiber tracts as predictors of tau-PET accumulation in a priori defined regions of interest (ROIs) and fiber-tract projection areas. In mediation analyses we tested whether tau-PET accumulation mediates the effect of florbetapir-PET in the whole WM on cognition. Finally, we assessed the role of myelin alteration on the association between APOE and tau-PET accumulation.

**Results:**

Lower florbetapir-PET in the whole WM or at a given fiber tract was predictive of faster tau-PET accumulation in Braak stages or the connected grey matter areas in Aβ+ participants. Faster tau-PET accumulation in higher cortical brain areas mediated the association between a decrease in florbetapir-PET in the WM and a faster rate of decline in global cognition and episodic memory. APOE ε4 genotype was associated with a worse decrease in the whole WM florbetapir-PET and thus enhanced tau-PET accumulation.

**Conclusion:**

Myelin alterations are associated in an APOE ε4 dependent manner with faster tau progression and cognitive decline, and may therefore play a role in the etiology of AD.

**Supplementary Information:**

The online version contains supplementary material available at 10.1007/s00259-023-06530-8.

## Introduction

Alzheimer’s disease (AD) is the major cause of age-related dementia. The disease defining pathologies are beta-amyloid (Aβ) plaques and neurofibrillary tau tangles. In particular, fibrillar tau is associated with neurodegeneration and cognitive decline [1], and is thus a key pathology driving clinical progression. During the course of AD, fibrillar tau preferentially progresses between connected brain regions [2, 3], suggesting that tau spreads along axonal connections [4, 5]. The spatial pattern of regional tau progression resembles the level of myelination of the connecting fibers, where regions connected by ontogenetically lower myelinated fiber tracts show higher susceptibility to tau deposition [6, 7]. These findings suggest that lower myelination may provide a vulnerability factor to the development of tau pathology in AD.

In patients with AD, myelin in the white matter (WM) is reduced as detected by single-cell transcriptomics of myelinating oligodendrocytes [8, 9], histochemical staining [8–10], and neuroimaging [11–13] of myelin alterations. In transgenic mouse models of Aβ and tau pathology, myelin alterations can be observed before the overt occurrence of amyloid plaques and fibrillar tau [14, 15]. Taken together, these observations raise the possibility that myelin alterations are associated with the formation of fibrillar tau in AD [16, 17]. However, it remains to be addressed whether in patients with AD, a decrease in myelin is associated with the progression of tau deposition. Therefore, our first major aim was to test the association between lower degree of myelin in fiber tracts and higher rates of tau-accumulation in the connected grey matter (GM) areas. Motivated by previous observations that those regions connected by ontogenetically lower myelinated fiber tracts are more susceptible to tau accumulation in AD [6, 7], our auxiliary aim was to test whether any association between myelin impairment and tau accumulation in patients with AD is particularly pronounced for typically lower myelinated fiber tracts.

In order to address these aims, we combined florbetapir-PET for the measurement of myelin with longitudinal assessment of fibrillar tau accumulation in a sample of clinically well-characterized patients with biomarker evidence of AD [18]. Florbetapir-PET was originally developed for the assessment of amyloid plaques [19], but — among other amyloid-sensitive PET tracers — has been recently repurposed for the assessment of myelin in the brain [20]. Amyloid-PET tracers may bind not only to the beta-sheet structure of fibrillar Aβ, but also to that of myelin-binding protein [21], enabling the quantification of myelin in the brain [22].

Our second major aim was to test whether the association between WM myelin and tau pathology is modulated by the presence of the APOE ε4 allele, i.e., the most important genetic risk factor of AD [23, 24]. The rationale for this aim is that APOE is the main transporter protein of cholesterol [25], i.e., a major component of myelin [26]. The APOE ε4 polymorphism was associated with reduced cholesterol biosynthesis in myelinating oligodendrocytes [27] and accelerated myelin reduction in normal aging [28, 29]. APOE ε4 expression in a transgenic mouse model of AD was associated with a substantial loss of myelin and increased tau pathology [30], suggesting that APOE ε4 is linked to tau pathology potentially via myelin alterations. In humans, APOE ε4 was strongly associated with increased levels of amyloid deposition in the brain [31]; however, APOE ε4 was also associated with higher tau accumulation independently from its effect on amyloid deposition in AD [32]. Therefore, our aim was to test whether myelin alterations assessed by florbetapir PET interact with APOE ε4 status to influence the rate of tau-PET increase.

## Methods

### ADNI participants

We included a sample of 151 participants from the Alzheimer’s Disease Neuroimaging Initiative (ADNI; http://adni.loni.usc.edu/), including a control group with 43 CN Aβ− participants and 108 participants within the AD spectrum consisting of 56 CN Aβ+, 32 MCI Aβ+, and 20 Aβ+ AD dementia. ADNI is a prospective multicenter study on biomarker and neuroimaging changes in AD [18]. The inclusion criteria for the current study beyond those of ADNI were the availability of T1-weighted MRI, FLAIR, [^18^F]-florbetapir amyloid-PET, at least 2 measures of [^18^F]-flortaucipir tau-PET (for follow-up duration see Table 1) and cognitive measures. In addition, participants were selected based on the inclusion in either a control group of CN Aβ− or AD spectrum group with abnormally elevated amyloid deposition (Aβ+). The Aβ+ was defined as a global standardized uptake value ratio (SUVR) cutoff > 1.11 for [^18^F]-florbetapir-PET as established previously [33]. Participants were clinically diagnosed as cognitively normal (CN, Mini-Mental State Exam (MMSE) > 24, Clinical Dementia Rating (CDR) = 0, nondepressed), mildly cognitively impaired (MCI, MMSE > 24, CDR = 0.5, objective memory loss on the education adjusted Wechsler Memory Scale II, preserved activities of daily living), or demented (AD, MMSE of 20 to 26, CDR > 0.5, NINCDS/ADRDA criteria for probable AD).

**Table 1:** Sample characteristics

	Control	AD spectrum		
	CN Aβ− (*n*=43)	CN Aβ+ (*n*=56)	MCI Aβ+ (*n*=32)	AD Aβ+ (*n*=20)
Age, year	72.6 (7.1)	76.2 (7.0)	76.1 (6.7)	77.5 (9.9)
Sex (M/F)	18M/25F	23M/33F	17M/15F	11M/9F
Education, year	16.5 (2.4)	16.7 (2.2)	16.7 (2.5)	14.6 (2.3)
ADAS13 score	7.2 (2.9)	8.8 (5.2)	17.2 (6.6)	30.7 (9.8)
ADNI-MEM score	1.2 (0.5)	1.0 (0.7)	0.0 (0.6)	-0.9 (0.6)
APOE ε4 carriers −/+^a^	30-/13+	26-/30+	7-/24+	11-/8+
Global AV45-PET SUVR	1.0 (0.0)	1.3 (0.2)	1.4 (0.1)	1.5 (0.2)
Global tau-PET SUVR^b^	0.9 (0.0)	0.9 (0.1)	1.0 (0.1)	1.0 (0.2)
Tau-PET follow-up time, year	2.7 (1.3)	2.3 (1.2)	1.9 (1.0)	1.7 (0.7)
WMH volume (ml)^c^	2.4 (3.4)	6.2 (9.8)	11.0 (24.8)	14.0 (28.3)
Global WM SUVR^c^	2.1 (0.2)	2.3 (0.2)	2.1 (0.3)	2.1 (0.2)

*Aβ* amyloid-beta, *AD* Alzheimer’s disease, *APOE* apolipoprotein E, *CN* cognitive normal, *F* female, *M* male, *MCI* mild cognitive impairment, *MMSE* Mini-Mental State Exam

^a^APOE status is missing for one MCI and one AD dementia participants. The mean and standard deviation (in brackets) are shown for each continuous variable

^b^Global Tau-PET at baseline, using eroded WM as reference region

^c^Raw, non-transformed data

Ethical approval was obtained by the ADNI investigators at each participating ADNI site. All participants provided written informed consent.

### MRI acquisition and processing

MRI scans were performed on different 3T MRI scanners using standardized scanning protocols (detailed scan protocols can be found on https://adni.loni.usc.edu/wp-content/uploads/2017/07/ADNI3-MRI-protocols.pdf). T1w images were acquired using a 3D MPRAGE sequence with 1 mm isotropic voxel-size and a TR = 2300 ms. Fluid-attenuated inversion recovery (FLAIR) images were acquired using a 3D FLAIR sequence with 1.2×1×1 mm voxel-size and TR = 4800 ms.

Using the Advanced Normalization Tools (ANTs) longitudinal cortical thickness pipeline [34, 35], T1-weighted images underwent bias field correction, followed by brain extraction and non-linear normalization to MNI space through a high-dimensional warping algorithm [34, 35]. Using the estimated normalization parameters, we further transformed the Desikan-Killiany atlas (Desikan et al., 2006) and the reference regions for intensity normalization of PET images from MNI space to native space.

### PET acquisition and processing

Tau-PET was recorded in 6×5 min frames, 75-105 min post-injection of [^18^F]flortaucipir. Amyloid-PET was recorded in 4 × 5 min frames, 50–70 min post injection of [^18^F]florbetapir. PET images were realigned, averaged, and further standardized with respect to the orientation, voxel size and intensity by the ADNI PET core [36].

Tau-PET images were rigidly co-registered to the participant’s T1-weighted image and Tau-PET SUVRs were computed by normalizing the tau-PET images to the mean tau-PET tracer uptake of the eroded white matter reference region, based on recent recommendations for longitudinal tau-PET assessments [37, 38]. Because tau-PET uptake in the white matter reference region can be altered in AD [39], we also computed tau-PET SUVRs using the inferior cerebellar grey as an alternate reference region [37]. ROI-level tau-PET SUVRs were computed for three a priori established composite regions including Braak 1 (entorhinal), Braak 3+4 (limbic) and Braak 5+6 (neocortical), as defined by the Braak post-mortem staging of tau pathology [40]. The Braak-stage 2 region (hippocampus) was not included due to potential spill-in from known off-target binding of the flortaucipir tracer in the choroid plexus [41]. In addition, global tau-PET SUVRs were determined as the average of neocortical tau-PET SUVRs across multiple cortical regions within temporal, parietal, and frontal lobes as specified previously by us [42]. Tau-PET SUVR measures were log-transformed prior to analysis to approximate a normal distribution.

## Myelin in white matter

### Assessment of global myelin

The florbetapir-PET images were acquired up to 1 year spaced apart from the first tau-PET scan and cognitive assessment. In order to derive masks to compute global white matter (WM) measures of florbetapir-PET, we first segmented the T1-weighted images using SynthSeg [43]. For each participant, we generated WM and GM masks in native space by thresholding and binarizing the estimated tissue segmentation at a threshold of 0.5 for WM to be consistent with a previous publication using florbetapir-PET signal as a proxy of myelin [13]and a threshold of 0.3 for GM which is commonly used and is consistent with our previous publications [7, 44]. We eroded the WM mask so that voxels within 1-mm distance from any non-WM voxel were excluded. In addition, for each participant, we derived a normal appearing white matter (NAWM) mask and white matter hyperintensities (WMH) mask.

WMH were segmented using a fully automated, deep learning algorithm based on multi-dimensional gated recurrent units [45] (https://github.com/miac-research/mdgru) with 3D FLAIR and 3D T1w images as inputs. Small WMH clusters with fewer than 5 voxels were excluded. For each participant, we generated NAWM masks by subtracting the WMH binary masks from the WM masks.

Florbetapir-PET images were rigidly co-registered to the participant’s T1-weighted images in native space and intensity normalized with the cerebellar grey as the reference regions, resulting in SUVR values [13]. Finally, using the individual WM, NAWM, WMH, or GM masks we computed the median florbetapir-PET SUVR values within each mask for each individual. For all further SUVRs calculations, we have used the median and not the mean as the median is more robust to outliers.

## Assessment of regional fiber-tract myelin and tau-PET in projection zones

Next, we extracted florbetapir-PET SUVRs from fiber-tract ROIs. To this end, we employed an unbiased fiber orientation distribution (FOD) template using a similar approach as previously described [46]. Specifically, we analyzed multi-shell diffusion data from 45 individuals included in ADNI across the AD continuum and performed multi-tissue constrained spherical deconvolution to build the FOD template. We applied TractSeg, a deep learning-based method for automated white matter bundle segmentation, based on the FOD template to construct 72 anatomically well-established white matter fiber tracts [47]. We excluded fiber tracts located in the cerebellum and the pons as we were interested in tracts with cortical projections. In addition, we excluded the fornix due to CSF partial-volume effects that rendered the tracking unreliable. This procedure resulted in a final sample of 58 white matter fiber tracts (see supplementary Table 1 for a list of fiber tracts included in the current study).

We estimated the normalization parameters from T1-weighted images to FOD template space, using non-linear spatial normalization with ANTs [34]. Florbetapir-PET SUVR images were then spatially normalized to FOD template space using the ANTs-derived normalization parameters. We then derived median florbetapir-PET SUVRs from each of the 58 white-matter fiber tracts for each participant, by superimposing the florbetapir-PET SUVR images onto the fiber tracts and sampling the voxels along the streamlines using MRtrix3 [48].

To assess regional associations between tract-specific florbetapir-PET SUVRs and regional tau pathology, we determined regional tau-PET SUVRs in cortical GM projection areas of each of the fiber tracts. To this end, we used masks from the beginning and ending of the fiber tracts as obtained by TractSeg [47]. Using the ANT-derived normalization parameters, we spatially normalized the cortical GM projection area masks to MNI space. The spatially normalized masks were further masked with a cortical GM mask and applied to the tau-PET SUVR images in MNI space to extract the median regional tau-PET SUVRs in each cortical GM projection area.

Finally, in order to assess the normal myelin levels in cognitively normal individuals for the 58 white-matter fiber tracts, we employed a myelin water fraction (MWF) template derived from young to middle-age healthy adults (mean age = 25 years) [49]. We spatially normalized the MWF template from MNI space to FOD template space using the ANTs-derived normalization parameters. We then derived median MWF levels along each of the fiber tracts, by superimposing the MWF template onto the fiber tracts and sampling the voxels along the streamlines using MRtrix3 [48]. The tract-specific effect size of the associations between florbetapir-PET SUVR in the fiber tracts and tau-PET SUVR changes in the tract’s projection zones in the AD spectrum group were projected onto the fiber-tract map of MWF to test the hypothesis that late-developing normally lower-myelinated fiber tracts (as assessed in the healthy individuals) are prone to exhibit a stronger effect of myelin alterations on tau deposition in AD.

### APOE genotyping

APOE allele counts were provided by ADNI, and participants were classified as APOE ε4 carriers when at least one ε4 allele was detected, otherwise participants were classified as APOE ε4 non-carriers. In addition, we calculated a neuropathology-based weighted risk score for APOE (APOE-npscore) as previously described [50]. Briefly, to generate the APOE-npscore we weighted the different allele combinations (including ε2, ε3, ε4 alleles) by the natural log (ln) transformed odds ratios of the association of each allele combination with the risk of brain-autopsy confirmed cases with AD [51]. This yields a neuropathology-validated pseudo-continuous APOE risk score that was previously shown to be more sensitive to predict AD progression compared to alternative forms of APOE scores, such as the binary classification into APOE ε4 carriers vs non-carriers [50].

### Neuropsychological measures

To assess global cognition we used the extended Alzheimer’s Disease Assessment Scale (ADAS13), which is an extension of the 11-item cognitive subscale of the ADAS [52], including an additional test of delayed word recall and number cancellation [53]. To assess memory performance we used the pre-established composite memory score ADNI-MEM [54], which includes the Rey Auditory Verbal Learning Test, the ADAS, the Wechsler Logical Memory I and II, and the word recall of the MMSE [54].

## Statistical analysis

### Adjustment of florbetapir-PET SUVRs

Our main predictor variable was florbetapir-PET SUVR in the WM as a measure of myelin. In order to reduce any influence of florbetapir binding to amyloid-plaques in the GM on the florbetapir binding in the WM, we adjusted the WM florbetapir SUVR as previously described [13]. Briefly, we fitted a linear regression model with the global GM florbetapir-PET SUVR as the predictor and the global florbetapir-PET SUVR in the WM as the dependent variable in the CN group including both Aβ+ and Aβ- participants. We then adjusted the florbetapir-PET SUVRs in the WM for the GM florbetapir-PET signal by subtracting the predicted SUVRs (using the estimated linear models) from each observed global SUVR in the WM. Furthermore, in order to quantify to what extent, the florbetapir-PET SUVR in the WM of the symptomatic Aβ+ participants deviate from those in the CN group, we computed *z* scores of WM florbetapir-PET SUVRs, using the CN group as a reference. For sensitivity analyses we computed florbetapir *z* scores also for NAWM and WMH (for distribution see Supplementary Figure 1). For fiber-tract level analyses, we performed the same procedure using fiber tract-specific florbetapir-PET SUVRs as the dependent variables. All subsequent analyses were conducted based on the florbetapir *z* scores in the WM.

### Association between florbetapir z scores in the WM and tau accumulation rates

In our main analysis, we tested whether a decrease in myelin levels in the WM is associated with higher rates of change in tau-PET. To this end, we first determined the subject-level annual rate of change in tau-PET, using a previously established approach [55]. We fitted linear mixed effects models with tau-PET SUVR as the dependent variable, time from baseline as the independent variable, with random slope and intercept. Using the thus estimated rates of change of tau-PET as the dependent variables, we tested in a linear regression analyses the global florbetapir *z* scores in the WM as the predictor. In sensitivity analyses, we tested whether global florbetapir *z* score alterations in areas of WMH are driving the results. We thus repeated the regression analyses, this time using florbetapir *z* scores within either the NAWM or WMH as predictors of the rates of change of tau-PET.

### Association between florbetapir z scores in the WM and cognitive decline & mediation analysis

In order to assess whether a decrease in myelin levels in the WM is associated with faster cognitive decline, we calculated the rate of change in cognitive measures (including composite scores ADNI-MEM and ADAS13) using linear-mixed effect models as mentioned above. Using linear regression, we tested global florbetapir *z* scores in the WM as a predictor of change rate in cognitive measures. To test whether the association between myelin and changes in cognition were mediated via changes in tau-PET, we conducted mediation analyses. To that end we treated the global florbetapir *z* scores in the WM as the predictor, change rate in global tau-PET as a mediator, and ADNI-MEM or ADAS13 scores as outcomes. The significance of the mediation was assessed using 1000 bootstrapped iterations, as implemented in the “mediation” R package [56]. The effect size of the mediated effect was computed as the proportion of the average causal mediation effect to the total effects expressed as percentage [56].

### Association between fiber tract-level florbetapir z scores and tau-PET accumulation in connected brain areas

In the next step, we assessed the regional associations between fiber tract-specific myelin and tau-PET changes in projection areas. To that end, for each fiber tract, we first computed the association between fiber tract-specific florbetapir *z* scores and tau-PET changes in the connected areas for each fiber tract, and the resulting distribution of *β*-values was tested against zero, using a one-sample *t*-test. Next, in order to test the hypothesis that the association between myelin alterations and tau change is particularly pronounced in ontogenetically less myelinated fiber tracts, we computed a spatial correlation between the fiber-tract *β*-values from the fiber tract-specific regressions and the fiber tract-specific MWF values from the MWF template of healthy individuals [49].

### The effect of APOE ε4 on the association between florbetapir z scores in the WM and tau-PET accumulation

Finally, we assessed the role of APOE using an ANCOVA analysis where APOE ε4 status was tested as a predictor of global florbetapir *z* scores in the WM or tau-PET changes. To test whether the association between APOE ε4 status and tau-PET changes is mediated via global florbetapir *z* scores in WM, we conducted a mediation analysis. To that end we treated the APOE ε4 status as the predictor, global florbetapir *z* scores in WM as a mediator, and tau-PET changes as the outcome. We next tested whether APOE ε4 status modulates the association between myelin and tau change by testing the interaction APOE ε4 status by global florbetapir *z* scores in the WM on tau-PET changes.

All above-mentioned models were controlled for age, sex, education, diagnosis, cortical florbetapir-PET SUVR, maximum follow-up duration, and time difference between florbetapir scan and tau-PET/cognitive measures. In addition, as sensitivity analyses to control for baseline severity of tau pathology, we controlled all above-mentioned models for the global tau-PET levels at baseline. All statistical analyses were performed using R statistical software (http://www.R-project.org). P-values were considered significant when meeting the *α*-threshold of 0.05. In the current study we chose not to implement correction for multiple comparisons in accordance with statistical guidelines that advise against utilizing correction for multiple comparisons in studies with a limited number of planned comparisons and are hypothesis driven [57].

## Results

We included a total of 108 participants on the AD continuum (Aβ+ CN/MC/AD) and 43 control participants (Aβ- CN) with longitudinal tau-PET and a baseline florbetapir-PET measure. Sample characteristics are shown in Table 1.

### Lower florbetapir PET in the WM is associated with higher tau accumulation rates

**Fig. 1:**
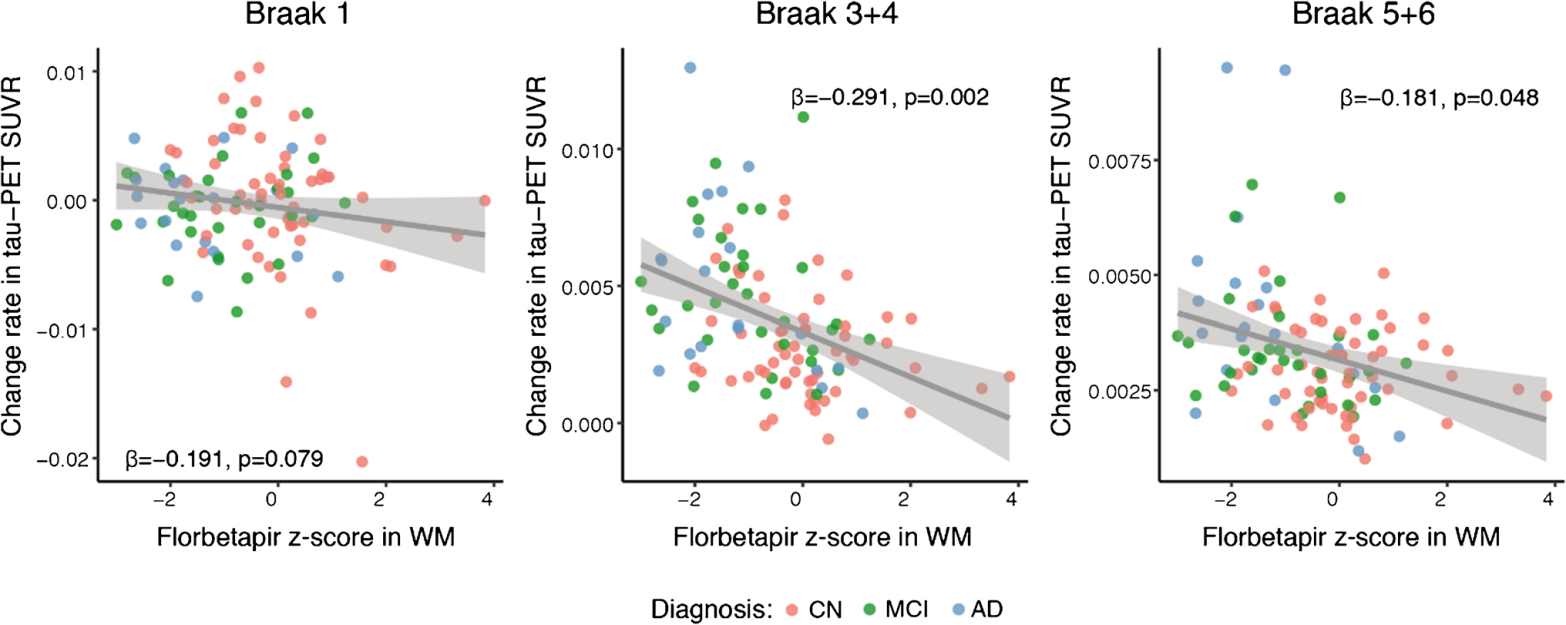
Association between florbetapir *z* scores in WM and change rate in tau-PET SUVRs. The scatterplots show the associations between florbetapir *z* scores in WM and change rate in tau-PET SUVRs for Aβ+ participants. Observations are color-coded by diagnosis and standardized *β-*values with *p*-values are displayed. AD = Alzheimer’s disease; CN = Cognitive normal; MCI = Mild cognitive impairment; WM = White matter

First, we addressed our aim to test whether a decrease in myelin levels in the WM is associated with higher tau-PET accumulation in participants within the AD spectrum (Aβ+ participants, Table 1). We found that lower florbetapir *z* scores in the global WM were significantly associated with higher rates of subsequent tau-PET accumulation in higher cortical areas among Aβ+ participants (Braak 3+4: *β*=−0.291, *p*=0.002; Braak 5+6: *β*=−0.181, *p*=0.048; Fig. 1) but not in the entorhinal cortex (Braak 1, *β*=−0.191, *p*=0.079; Fig. 1). Note that these and all subsequent analyses were controlled for florbetapir binding in the GM — among other covariates — to partial out any influence of amyloid plaque deposition in the GM. As expected, no associations were observed in the control group consisting of cognitively normal (CN) participants without biomarker evidence of elevated Aβ deposition (CN Aβ−, Braak 1: *β*=−0.103, *p*=0.6; Braak 3+4: *β*=−0.157, *p*=0.4; Braak 5+6: *β*=−0.162, *p*=0.4). These results suggest that reduced myelin levels in the WM are associated with faster rates of tau accumulation in subjects with biomarker evidence of AD.

When controlling the analyses for baseline global tau-PET levels, we confirmed a significant association between the florbetapir *z* scores in the WM and the rate of change of tau-PET in Braak stage 1 (*β*=−0.219, *p* = 0.049) and Braak stage 3+4 (*β*=−0.124, *p* = 0.036).

As sensitivity analyses, we repeated these analyses using a cerebellar reference region instead of the white matter reference region for the calculation of tau-PET SUVRs. We found consistent results where lower florbetapir *z* scores in the global WM were significantly associated with higher rates of subsequent tau-PET accumulation in all Braak stages among Aβ+ participants (Braak 1: *β*=−0.258, *p*=0.018, Braak 3+4: *β*=−0.309, *p*=0.0008; Braak 5+6: *β*=−0.227, *p*=0.011; Supplementary Figure 1).

Florbetapir uptake was significantly reduced in WMH areas compared to those within the NAWM (*t*(295)=7.024, *p* < 0.001), replicating previous findings [13]. Next, we tested whether the observed association between florbetapir *z* scores in the WM and tau-PET accumulation depends on the presence of WMH. To this end, we extracted the global florbetapir *z* scores in areas of WMH, or alternatively exclusively in the NAWM excluding WMH. Consistent with the results for the whole WM, lower global florbetapir *z* scores both in the WMH and NAWM were associated with higher tau-PET accumulation in higher cortical regions among the Aβ+ participants (Supplementary Figure 2), suggesting that the association between florbetapir *z* scores in the WM and tau-PET accumulation was not exclusively driven by WMH.

### Lower florbetapir PET in the WM was associated with cognitive decline via tau accumulation

**Fig. 2:**
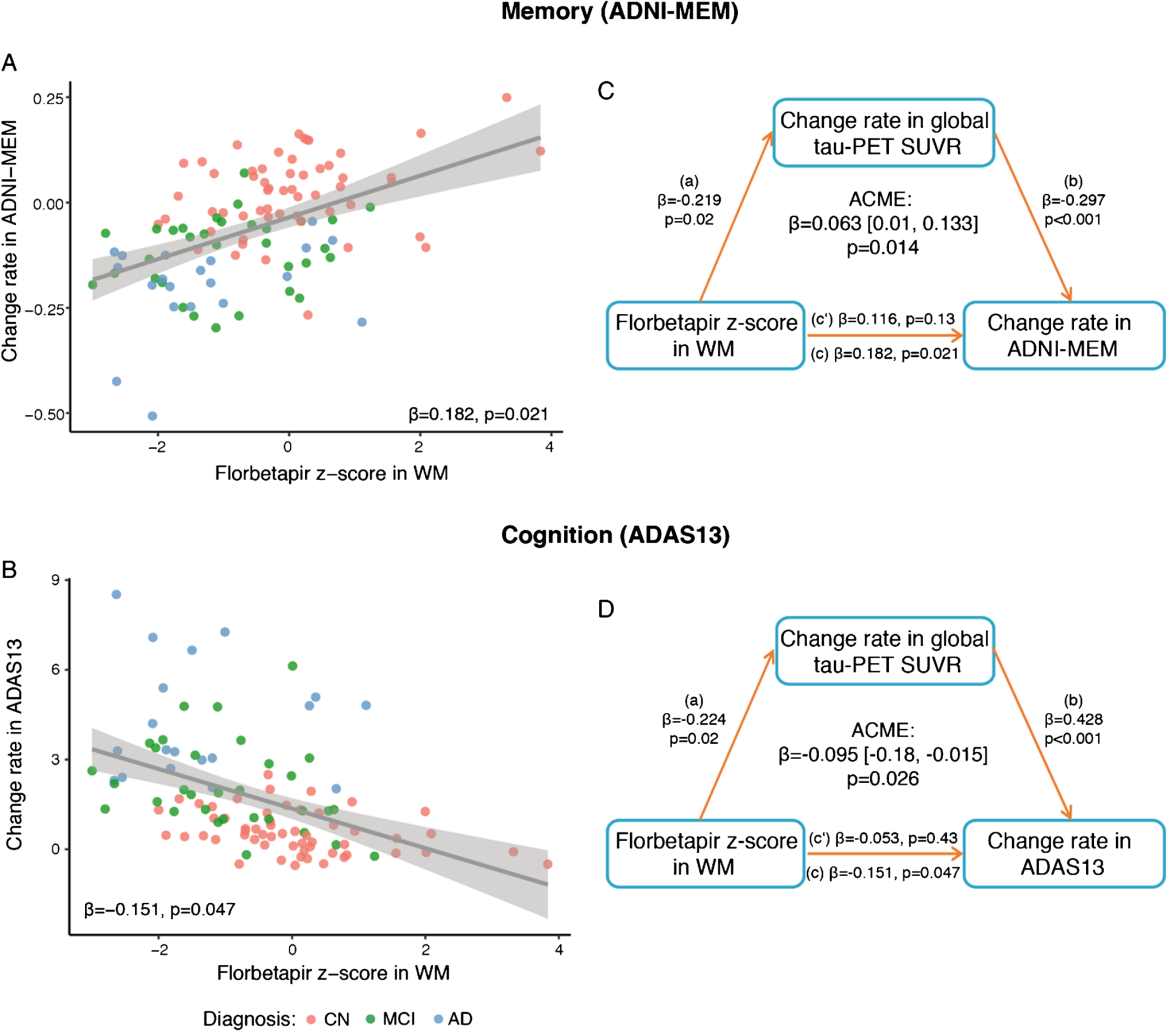
Tau-PET accumulation mediates the association between florbetapir *z* scores in WM and cognitive decline. **A**, **B** Scatterplots showing the association between florbetapir *z* scores in WM and change rate in memory (ADNI-MEM; **A**) or cognition (ADAS13; **B**). Observations are color coded by diagnosis and standardized *β*-values with *p*-values are displayed. **C, D** Mediation analyses showing that the association between florbetapir *z* scores in WM and changes in memory performance (**C**) or cognition (**D**) is mediated by the change rate in global tau-PET SUVR. Path values are displayed as *β*-values with *p*-values. The path weight c indicates the effect of florbetapir *z* scores in WM on changes in memory or cognition without taking change rate in global tau-PET SUVR into account, the path coefficient c’ indicates the corresponding effect of florbetapir *z* scores in WM after accounting for the mediator change rate in global tau-PET SUVR. Mediation effects were determined based on bootstrapping with 1000 iterations. All paths are controlled for age, sex, education, diagnosis, cortical florbetapir-PET SUVR, maximum follow-up duration, and time difference between florbetapir scan and cognitive measures. AD = Alzheimer’s disease; ADAS13 = Alzheimer’s Disease Assessment Scale cognitive subscale; ACME = Average causal mediation effect; ADNI-MEM = Alzheimer’s Disease Neuroimaging Initiative memory composite; CN = cognitive normal; MCI = mild cognitive impairment; WM = white matter

Next, we tested the association between the decrease in myelin and the rate of cognitive decline. We found that lower global florbetapir *z* scores in the WM were significantly associated with a faster decline in memory performance (ADNI-MEM: *β*=0.182, *p*=0.021; Fig. 2A) and global cognition (ADAS13: *β*=−0.151, *p*=0.047; Fig. 2B). Using bootstrapped mediation analyses, we found that higher rates of global tau-PET accumulation mediated the effect of lower global florbetapir *z* scores in the WM on the rate of change in memory (ADNI-MEM: β=0.063 [95% CI: 0.01, 0.133], *p*=0.014, proportion mediated = 33.8%; Fig. 2C) and global cognition (ADAS13: *β*=−0.095 [95% CI: −0.180, −0.015], *p*=0.026, proportion mediated = 63.8%; Fig. 2D), suggesting that the effect of myelin on tau explains the association between demyelination and faster cognitive decline.

### Fiber tract-level florbetapir PET was associated with tau-PET accumulation in connected brain areas

**Fig. 3:**
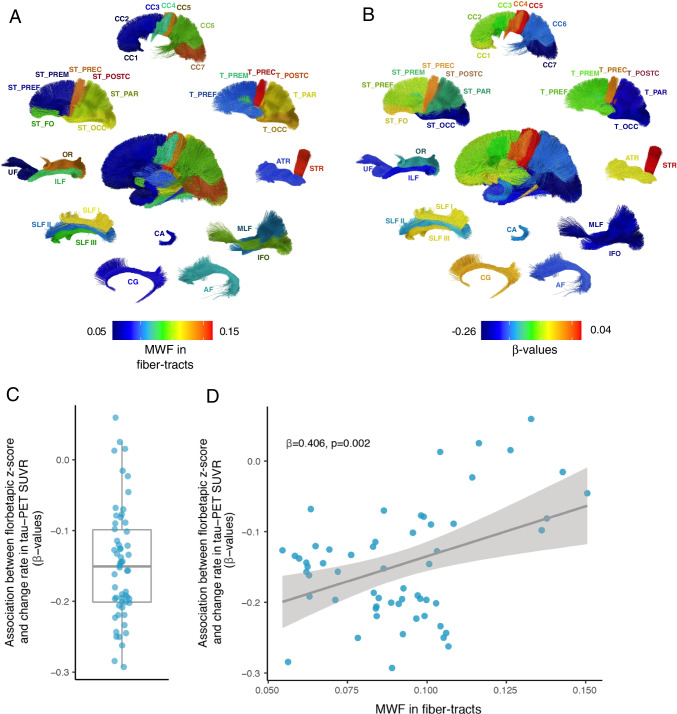
Association between florbetapir *z* scores in fiber tracts and change rate in tau-PET SUVRs in connected regions. **A** The myelin water fraction (MWF) quantifying the myelin levels for each major fiber tract in cognitively normal subjects. The color coding refers to the MWF level, with warmer colors corresponding to higher MWF levels. **B** The effect sizes (standardized *β*-value) from the linear regression analyses including fiber tract-level florbetapir *z* scores as a predictor of tau-PET in the connected cortical areas are plotted for each fiber tract. Warmer colors correspond to a stronger β-coefficient of the association between lower florbetapir *z* scores in a given fiber tract and higher rate of tau-PET increase in the connected cortical areas. **C** Boxplot showing the *β*-values derived from the correlation between florbetapir *z* scores in fiber tracts and change rate in tau-PET in connected regions. Each dot represents a specific fiber tract. **D** Scatterplot showing the association between normative MWF in fiber tracts derived from healthy participants (x-axis) and *β*-values derived from the correlation between florbetapir *z* scores in fiber tracts and change rate in tau-PET in connected regions. MWF = myelin water fraction

In the next step, we tested whether there is a spatial correspondence between fiber tract-level alterations in florbetapir *z* scores and tau-PET accumulation in the connected GM regions. To this end, we extracted the florbetapir *z* scores in each of the 58 major well-established fiber tracts and tau-PET change rates in each of the tracts’ cortical projection areas in each participant. For each fiber tract, we regressed the rate of tau accumulation in the tract’s projection area on the tract-level florbetapir *z* scores. In a second-level analysis, we found that the associations between the fiber tract-specific florbetapir *z* scores and change rate in tau-PET in projection areas were significant across fiber tracts (*t*(57)= −14.099, *p*<0.001; Fig. 3A).

Due to heterochronicity of the myelination of the brain during development, fiber tracts in the normal brain show substantial differences in the degree of myelin [58]. Given that we and others previously observed that brain regions connected by typically lower myelinated fiber tracts are more susceptible to accumulate tau pathology in AD [59], we tested here our auxiliary hypothesis that the association between myelin impairment and tau accumulation is particularly pronounced for typically lower myelinated fiber tracts, using a MRI-derived template of myelin in the normal brain [49]. We observed for those fiber tracts that are lower myelinated in the normal brain a stronger association between the florbetapir *z* scores and tau-PET increases in the connected GM areas (*β*=0.406, *p*=0.002; Fig. 3B), suggesting that the association between demyelination and tau accumulation is stronger for those fiber tracts that are typically lower myelinated in the brain.

When controlling for global tau-PET levels at baseline, we found that the associations between the fiber tract-specific florbetapir *z* scores and change rate in tau-PET in projection areas were significant across fiber tracts (*t*(57)= −3.671, *p*<0.001).

### APOE ε4 influences the association between florbetapir z score in the WM and tau-PET accumulation

**Fig. 4:**
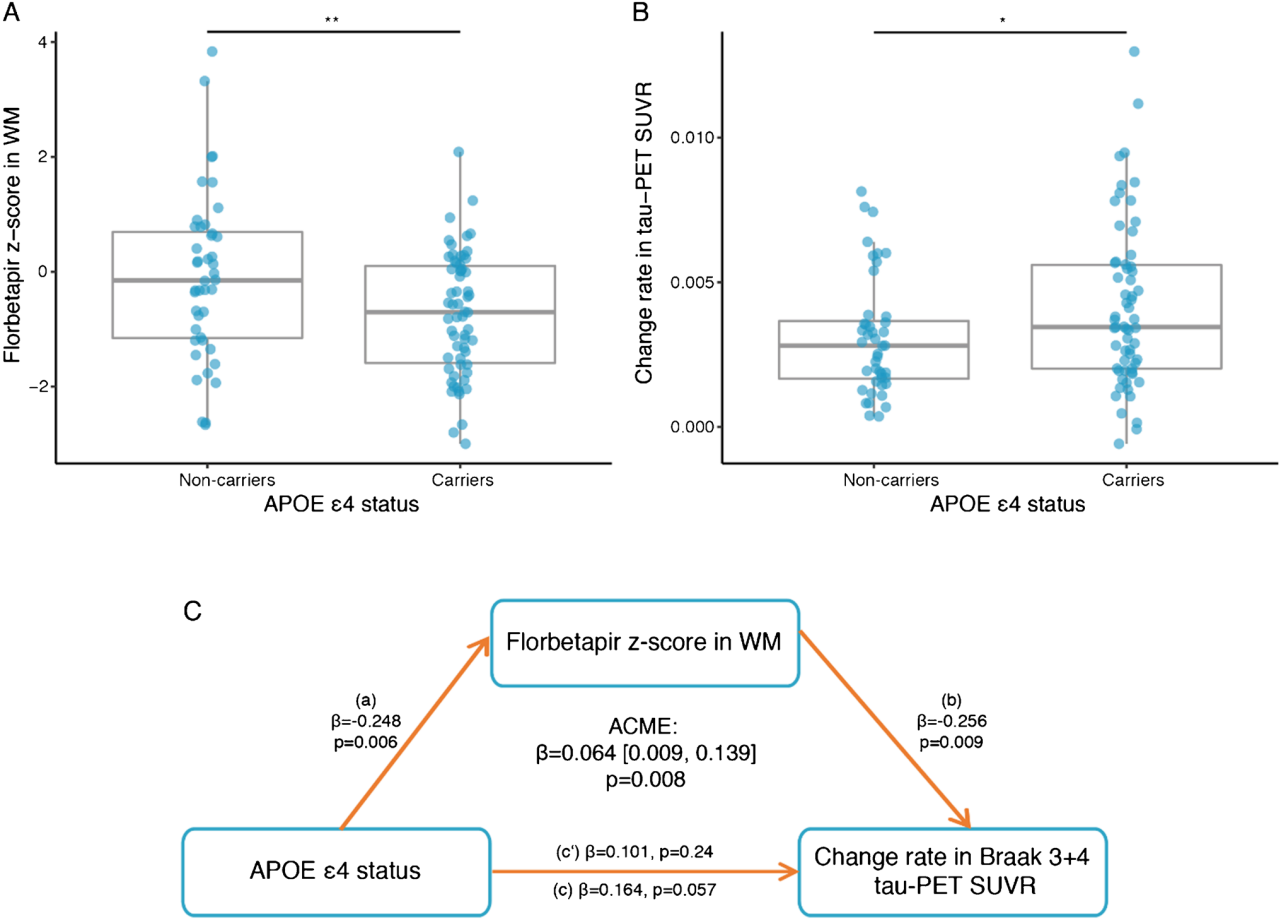
Florbetapir *z* scores mediate the effect of APOE ε4 status on change rate in tau-PET SUVRs. **A** Boxplot showing the effect of APOE ε4 status on florbetapir *z* scores in WM. **B** Boxplot showing the effect of APOE ε4 status on change rate in Braak 3+4 tau-PET SUVR. **C** Mediation analysis showing that the association between APOE ε4 status and change rate in Braak 3+4 tau-PET SUVR is mediated by florbetapir *z* scores in WM. Path values are displayed as *β*-values with *p*-values. The path weight c indicates the effect of APOE ε4 status on changes in tau-PET without taking florbetapir *z* score into account, the path coefficient c’ indicates the corresponding effect of APOE-npscore after accounting for the mediator including florbetapir *z* scores in WM. Mediation effect was determined based on bootstrapping with 1000 iterations. All paths are controlled for age, sex, education, diagnosis, cortical florbetapir-PET SUVR, maximum follow-up duration, and time difference between florbetapir scan and tau-PET scan. ACME = average causal mediation effect; APOE = apolipoprotein E; WM = white matter

For our second major aim, we tested the effect of APOE ε4 on both florbetapir *z* score and tau-PET accumulation. We found that florbetapir *z* scores in the WM were reduced in the APOE ε4 carriers compared to those in the APOE ε4 non-carriers within the Aβ+ group (*F*(1,99) = 8.622, 0.004; Fig. 4A). Furthermore, APOE ε4 carriers showed higher rates of tau-PET accumulation in Braak-stage 3+4 ROIs (*F*(1,97) = 5.942, 0.017; Fig. 4B).

**Fig. 5:**
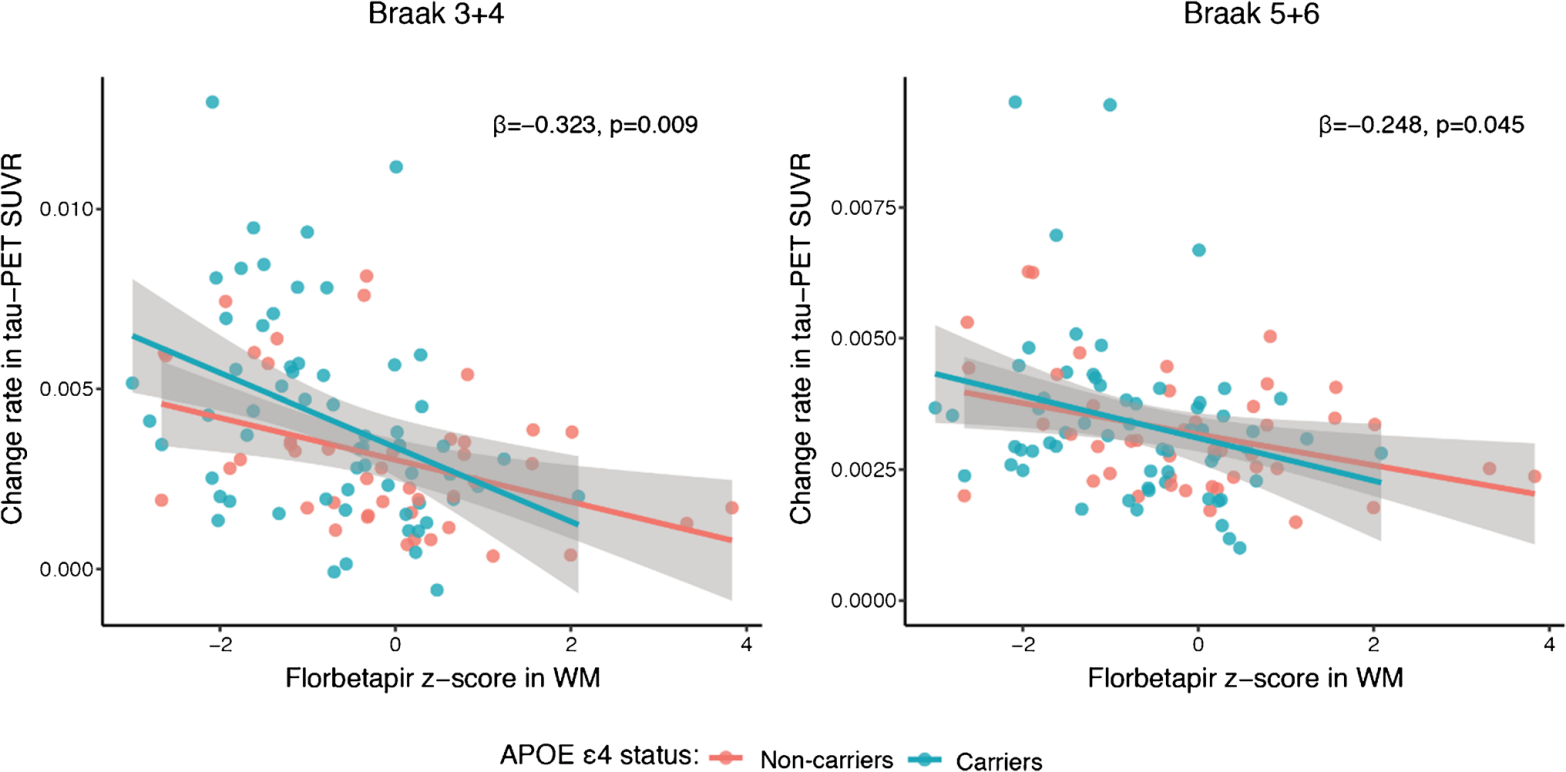
APOE ε4 status modulates the effect of florbetapir *z* scores on change rate in tau-PET SUVRs. Scatterplots showing the interaction between APOE ε4 status and global florbetapir *z* scores in the WM on change rate in tau-PET in Braak stages 3+4 and 5+6. Red line is the regression line for APOE ε4 non-carriers and the blue regression line is for APOE ε4 carriers

Next, we tested whether the effect of APOE ε4 status on tau-PET accumulation is mediated by global florbetapir *z* scores in the WM. Using bootstrapped mediation analysis, we found that global florbetapir *z* scores in the WM mediated the effect of APOE ε4 status on the rate of tau-PET accumulation in Braak 3+4 ROIs (*β*=0.064 [95% CI: 0.009, 0.140], *p*=0.008, proportion mediated = 39.3%; Fig. 4C). These results suggest that APOE ε4 is associated with tau accumulation through its effect on myelin impairment. In addition, we tested whether APOE ε4 status also worsens the association between myelin alterations and tau accumulation. In an interaction analysis, we found a significant interaction between APOE ε4 status and florbetapir *z* scores in the WM, where APOE ε4 carriers showed stronger association between lower global florbetapir *z* scores in the WM and higher tau-PET accumulation in Braak stage 3+4 ROIs (*β*=−0.323, *p*=0.009; Fig. 5A) and 5+6 ROIs (*β*=−0.248, *p*=0.045; Fig. 5A). When controlling for global-tau PET values, the interaction did not reach significance, probably due to limited power for testing interaction terms.

In order to ensure that analysis of the effects of the APOE e4 allele does not depend on the binary classification of the APOE ε4 status, we repeated the analysis based on a recently developed neuropathology-weighted measure of APOE gene dosage [60]. The result pattern remained the same (Supplementary results and Supplementary Figure 3), supporting the robustness of our analysis.

## Discussion

Myelin alterations frequently occur in AD [16] and are exacerbated in APOE ε4 carriers [29, 61], but the association between APOE ε4, myelin alterations, and primary AD pathology remains unclear [62]. By repurposing the florbetpir-PET tracer to measure myelin in the WM, we found that decreased florbetapir *z* scores in the WM were associated with faster rates of cortical tau-PET accumulation, which mediated the effect on cognitive decline. APOE ε4 was associated with worse florbetapir *z* score reduction and interacted with florbetapir *z* scores in enhancing the rate of tau-PET accumulation, suggesting that APOE ε4 and myelin alterations show a synergistic effect on tau accumulation. Together, these results when controlled for amyloid levels in the GM suggest that a decrease in WM myelin is associated with accelerated fibrillar tau accumulation and thus cognitive decline, where the presence of APOE ε4 exacerbate the association between myelin loss and tau accumulation.

For our first major finding, we demonstrated that lower florbetapir *z* scores were associated with faster subsequent tau-PET accumulation in isocortical brain areas, suggesting that a decrease in myelin is predictive of faster tau accumulation in AD. Our results are in general agreement with previous neuroimaging and brain autopsy studies reporting reduced WM myelin in AD [12, 63–65]. Consistent with the current results, available evidence from previous studies suggest that myelin alterations occur early in the development of tau pathology: Lower MRI-assessed myelin water fraction in the WM was associated with higher CSF biomarker levels of phospho-tau in preclinical AD [12], and post-mortem assessed myelin-specific ceramide levels were reduced in early Braak-stage region of tau pathology including the medial temporal lobe [10]. Likewise, myelin changes emerged before overt deposition of fibrillar tau in a transgenic mouse model of tau pathology [14], providing experimental support for an early involvement of myelin alterations in the development of tau pathology. Our longitudinal tau-PET imaging study in patients with AD significantly advances these previous cross-sectional studies, demonstrating for the first time that myelin alterations are predictive of faster tau accumulation. While we caution that the current findings should not be interpreted in a causative manner, our findings suggest that myelin alterations may play a role in the etiology of tau pathology.

Furthermore, we demonstrated in our fiber tract-level analysis that the associations between fiber-tract myelin alterations on tau PET accumulation were strongest for those fiber tracts that are typically lower myelinated in the human brain. These findings are in agreement with previous observations of enhanced regional susceptibility to tau accumulation of those brain regions connected by lower myelinated fiber tracts [6, 7]. Furthermore, in humans, we and others previously showed that tau pathology preferentially progresses along closely connected brain regions in patients with AD [3, 4, 66], suggesting that interregional connections provide pathways for the progression of tau pathology in the brain [67, 68]. Therefore, one possibility is that myelin alterations, particularly in late-developing lower myelinated fiber tracts, may enhance the spreading of tau pathology. However, the exact molecular mechanisms need to be still deciphered.

Our second major finding suggests that myelin alteration play an important role in the association between APOE and tau pathology. Our mediation analysis suggested that myelin alterations contribute to the association between APOE ε4 and tau progression, suggesting that APOE and myelin alterations are part of a common pathomechanistic pathway linked to tau pathology. APOE ε4 was previously found to be associated with reduced cholesterol localization and homeostasis in myelinating oligodendrocytes [27]. In transgenic mouse models of tau pathology, myelin was impaired [69, 70], and expression of human APOE ε4 was associated with both higher levels of myelin damage and tau pathology [30, 70]. These studies substantiate a link between APOE ε4, myelin alterations, and tau pathology. Furthermore, we found that in APOE ε4 carriers, the association between myelin alterations and tau accumulation was pronounced. A potential pathomechanism underlying this interaction between APOE ε4 and myelin is that microglial phagocytosis of cholesterol-rich lipid droplets from impaired myelin may lead to microglial senescence [71, 72] which is exacerbated by microglial APOE ε4 expression [66], rendering microglia less efficient in phagocyting core AD pathologies [67]. Therefore, APOE ε4 may interact with myelin loss such that impaired microglial activation and increased release of inflammasome [70] enhance the accumulation of tau pathology in AD [68]. Our results encourage future experimental studies to uncover the molecular mechanisms that explain the association between APOE, myelin alterations and tau pathology. It should be also noted that consistent with previous findings [13], we observed worse florbetapir-PET signal loss in areas of WMH compared to NAWM. WMH may stem from small vessel disease related processes or, alternatively, relate to primary AD pathology [73, 74]. The disentanglement of the sources of WMH and associated myelin loss warrants further investigation.

In order to interpret the current findings, some caveats need to be taken into account. First, the florbetapir-PET tracer which was originally developed for the detection of amyloid plaques in the GM was repurposed as a measure of myelin in the WM in the current study. In AD the potential influence of amyloid plaque deposition on the florbetapir-PET binding in the WM is particularly pertinent. In order to mitigate any potential influence of binding to amyloid plaques in the GM, we adopted several steps including (1) the erosion of the WM in order to reduce any spill-over effects from GM regions, and (2) adjusting the florbetapir-PET WM signal for the florbetapir-PET GM signal, which rendered the WM and GM florbetapir signal uncorrelated and thus removed the influence of GM signal on the WM. Furthermore, histochemical brain autopsy results suggest that amyloid deposition occurs predominantly close to the WM border and vanishes rapidly within less than 1 mm [75], and may not account for amyloid-PET binding in the WM [76]. Eroding the WM border as implemented in our study may have effectively reduced any PET binding to amyloid in the WM. We note further that there is now solid evidence that amyloid-PET tracers bind to the beta-sheet structure of amyloid and the myelin binding protein in the WM [77, 78], supporting the validity of our approach. In conclusion, while the substrate of amyloid-PET tracer binding in the WM remains to be fully clarified and an influence of amyloid on WM binding cannot be excluded in AD, the current findings provide convincing evidence for myelin alterations assessed by florbetapir-PET binding in AD.

Another caveat is that we could not assess whether the observed effects differ by clinical disease stage due to limited sample size. In particular, in the early asymptomatic phase of AD, any myelin reductions and tau-PET increases are more limited and thus will require larger future studies. Lastly, we caution that most participants were highly educated individuals of Caucasian background with limited cerebrovascular disease. Therefore, the current results remain to be replicated in a group of individuals with a more heterogeneous socio-economic and cultural background. We further note that future studies may investigate the association between myelin alterations and the development of the deposition of amyloid plaques, which we could not test in the current study due to the necessity to correct the florbetapir-PET signal in the WM for the GM signal. Our results therefore suggest that the association between myelin alterations and tau pathology hold when controlling for amyloid GM levels. Yet, our results do not preclude that there is also an association between myelin alterations and amyloid plaque deposition[67, 79], which may synergistically influence the deposition of fibrillar tau. Lastly, we note the difficulty of disentangling effects on the cumulative level of tau-PET and the rates of tau-PET accumulation, given that both are intrinsically linked. In the current study we focused on the rates of tau-accumulation to model differences in the intra-individual increase in tau accumulation.

In summary, the current study provides to our best knowledge the first evidence for the association between APOE genotype, myelin alterations, and tau progression in AD. Myelin is a druggable target, and several already FDA-approved drugs such as clemastine, fingolimod, and rolipram could be potentially repurposed for enhancing myelination and thus slowing AD progression and cognitive decline [80]. Therefore, it is pivotal to better understand the association between myelin alterations and the formation of primary AD pathologies in AD to pave the way for drug interventions that may complement anti-amyloid drugs.

### Supplementary Information

Below is the link to the electronic supplementary material.

Supplementary file1 (DOCX 540 KB)

## Data Availability

Data used in this study are available from the ADNI database (adni.loni.usc.edu) upon registration and compliance with the data usage agreement.

## Code availability

The custom R scripting used for the analysis of the data will be made available upon reasonable request.
