# Supplementary material for: Florbetapir PET-assessed demyelination is associated with faster tau accumulation in an APOE ε4-dependent manner
Source: Eur J Nucl Med Mol Imaging. 2023 Dec 5;51(4):1035–49. doi: 10.1007/s00259-023-06530-8 (PMC10881623; doi:10.1007/s00259-023-06530-8)
Supplement: Supplementary file 1 — Supplementary file1 (DOCX 540 KB) [file 259_2023_6530_MOESM1_ESM.docx]

**Supplementary figures**


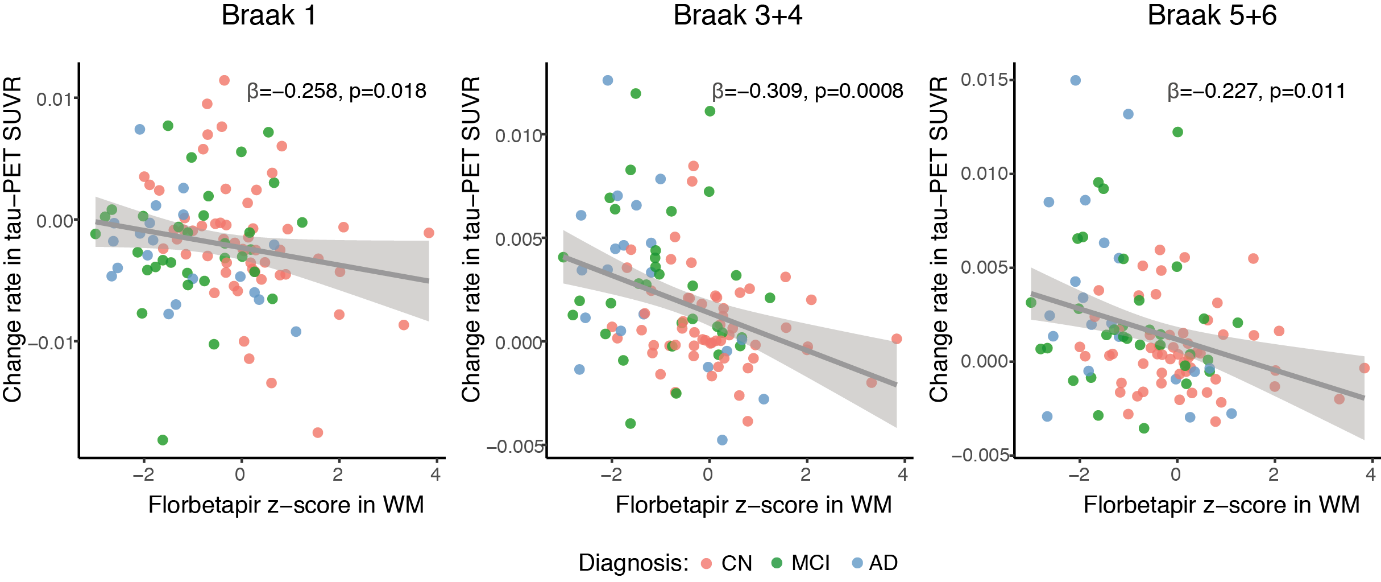


**Supplementary Figure 1: Association between florbetapir z-score in WM and change rate in tau-PET SUVRs calculated using a standard cerebellar reference region**

Scatterplots showing the association between florbetapir z-score in WM and change rate in tau-PET SUVRs for Aβ+ participants. Tau-PET SUVRs are calculated using a standard cerebellar reference region. Observations are color-coded by diagnosis and standardized β-values with p-values are displayed. AD = Alzheimer’s disease; CN = Cognitive normal; MCI = Mild cognitive impairment; WM = White matter.


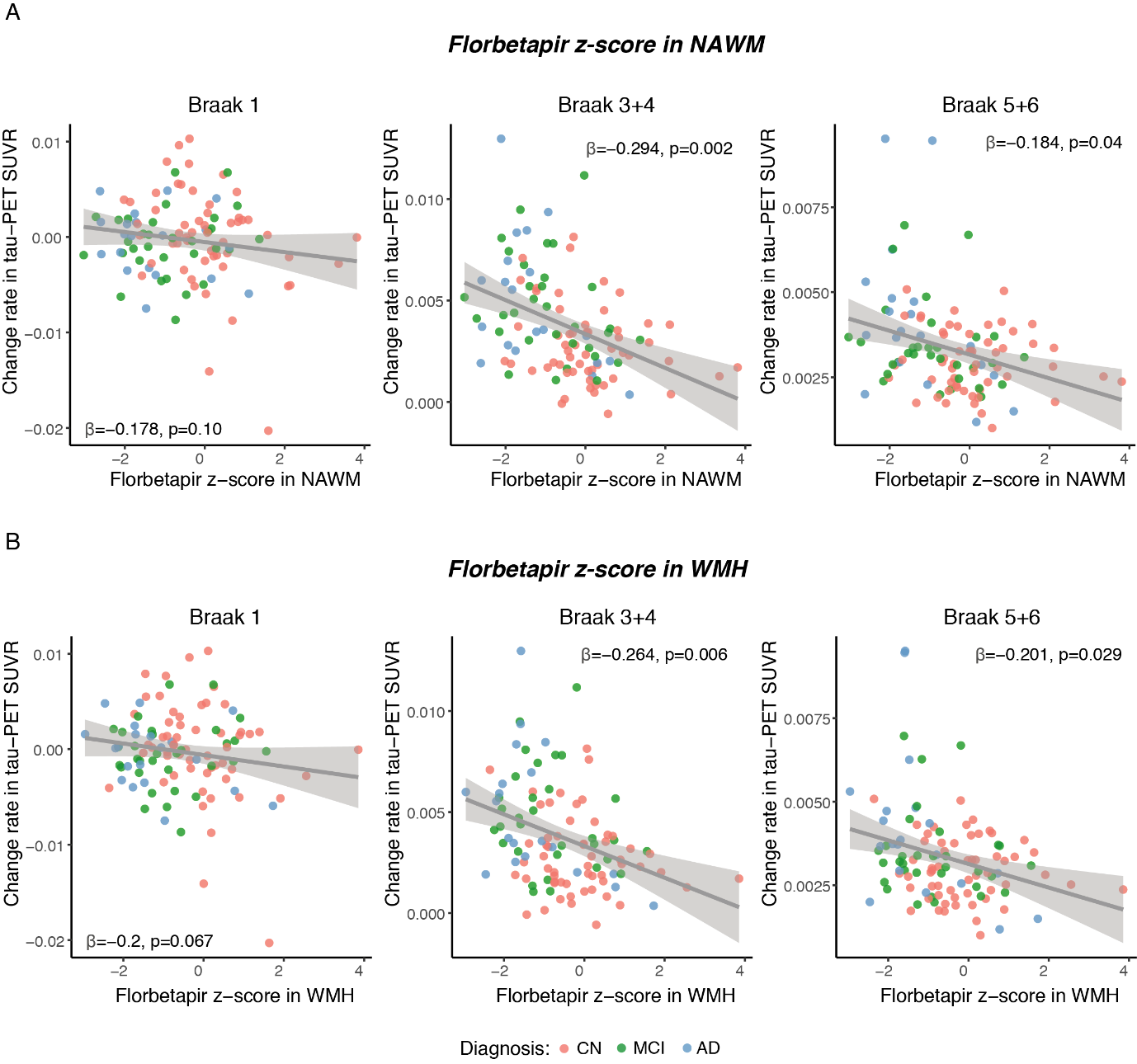


**Supplementary Figure 2: Association between florbetapir z-score in NAWM or WMH and change rate in tau-PET SUVRs**

Scatterplots showing the association between florbetapir z-score in NAWM (A) or WMH (B) and change rate in tau-PET SUVRs for Aβ+ participants. Observations are color coded by diagnosis and standardized β-values with p-values are displayed. AD = Alzheimer’s disease; CN = Cognitive normal; MCI = Mild cognitive impairment; NAWM = Normal appearing white matter; WM = White matter; WMH = White matter hyperintensities.

**
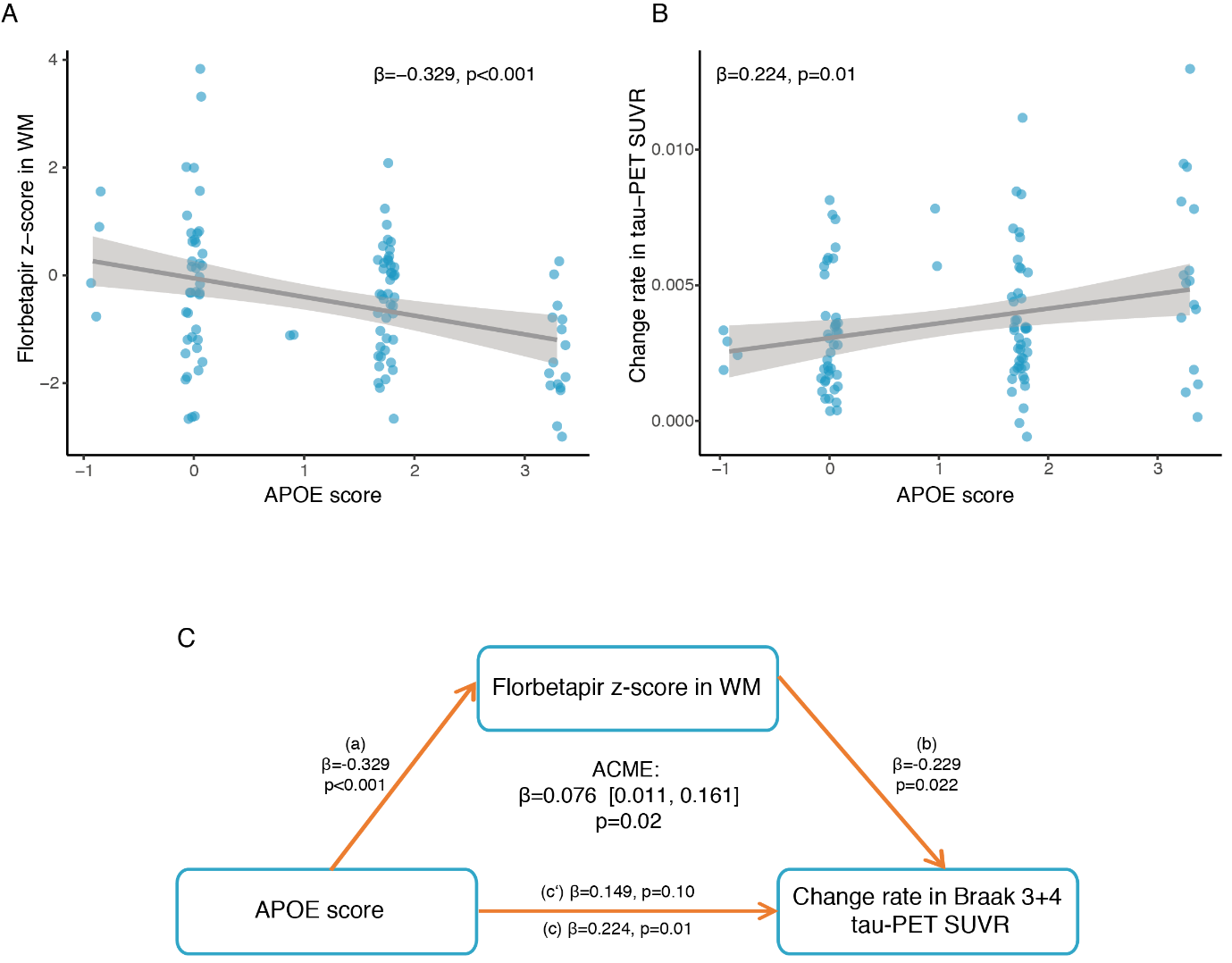
**

**Supplementary Figure 3: Florbetapir z-score mediates the effect of APOE-npscore on change rate in tau-PET SUVRs**

(A) Scatterplot showing the association between APOE-npscore and florbetapir z-score in WM. (B) Scatterplot showing the association between APOE-npscore and change rate in Braak 3+4 tau-PET SUVR. (C) Mediation analysis showing that the association between APOE-npscore and change rate in Braak 3+4 tau-PET SUVR is mediated by florbetapir z-score in WM. Path values are displayed as β-values with p-values. The path weight c indicates the effect of APOE-npscore on changes in tau-PET without taking florbetapir z-score into account, the path coefficient c’ indicates the corresponding effect of APOE-npscore after accounting for the mediator flobrbetapir z-score in WM. Mediation effect was determined based on bootstrapping with 1,000 iterations. All paths are controlled for age, sex, education, diagnosis, cortical florbetapir-PET SUVR, maximum follow-up duration, and time difference between florbetapir scan and tau-PET scan. ACME = Average causal mediation effect; APOE = Apolipoprotein E; WM = White matter.
